# The HaDREB2 transcription factor enhances basal thermotolerance and longevity of seeds through functional interaction with HaHSFA9

**DOI:** 10.1186/1471-2229-9-75

**Published:** 2009-06-19

**Authors:** Concepción Almoguera, Pilar Prieto-Dapena, Juan Díaz-Martín, José M Espinosa, Raúl Carranco, Juan Jordano

**Affiliations:** 1 Instituto de Recursos Naturales y Agrobiología de Sevilla, Consejo Superior de Investigaciones Científicas, Apartado 1052, 41080 Seville, Spain

## Abstract

**Background:**

Transcription factor HaDREB2 was identified in sunflower (*Helianthus annuus *L.) as a drought-responsive element-binding factor 2 (DREB2) with unique properties. HaDREB2 and the sunflower Heat Shock Factor A9 (HaHSFA9) co-activated the *Hahsp17.6G1 *promoter in sunflower embryos. Both factors could be involved in transcriptional co-activation of additional small heat stress protein (sHSP) promoters, and thus contribute to the HaHSFA9-mediated enhancement of longevity and basal thermotolerance of seeds.

**Results:**

We found that overexpression of HaDREB2 in seeds did not enhance longevity. This was deduced from assays of basal thermotolerance and controlled seed-deterioration, which were performed with transgenic tobacco. Furthermore, the constitutive overexpression of HaDREB2 did not increase thermotolerance in seedlings or result in the accumulation of HSPs at normal growth temperatures. In contrast, when HaDREB2 and HaHSFA9 were conjointly overexpressed in seeds, we observed positive effects on seed longevity, beyond those observed with overexpression of HaHSFA9 alone. Such additional effects are accompanied by a subtle enhancement of the accumulation of subsets of sHSPs belonging to the CI and CII cytosolic classes.

**Conclusion:**

Our results reveal the functional interdependency of HaDREB2 and HaHSFA9 in seeds. HaDREB2 differs from other previously characterized DREB2 factors in plants in terms of its unique functional interaction with the seed-specific HaHSFA9 factor. No functional interaction between HaDREB2 and HaHSFA9 was observed when both factors were conjointly overexpressed in vegetative tissues. We therefore suggest that additional, seed-specific factors, or protein modifications, could be required for the functional interaction between HaDREB2 and HaHSFA9.

## Background

HSFA9 is the sole plant HSF that has been found to be seed-specific [1,2]. Gain of function as a consequence of overexpression of HaHSFA9 in seeds showed the involvement of this transcription factor in basal thermotolerance and longevity. The transgenic seeds survive exposure to lethal temperatures after seed imbibition, without a previous, heat-acclimation treatment. The HaHSFA9 seeds also resist controlled deterioration procedures for rapid aging [3]. More recently, we have shown that ectopic overexpression of HaHSFA9 conferred dramatic resistance of vegetative tissues from young seedlings to severe dehydration, which was quantified as water loss of up to 98% of total water content [4]. In both cases HaHSFA9 activated a genetic program that includes subsets of HSPs normally expressed during zygotic embryogenesis in seeds; this program does not include late embryogenesis abundant (LEA) proteins. Furthermore, HaHSFA9 did not affect the accumulation of sucrose and raffinose oligosaccharides which, like LEA proteins might be also involved in seed longevity and desiccation tolerance (see e.g. [5-8] and other references discussed in [3,4]).

Our laboratory has been interested for a long time in the identification of additional transcription factors involved in controlling the "seed HSP program". HaHSFA9 and orthologous plant HSFs might be master regulators of such a program; our earlier work indicated that HaHSFA9 could be used to obtain partially improved phenotypes without producing any negative effects on plant growth, morphology or development, at least under laboratory conditions. Among candidate regulatory factors is HaDREB2, which is expressed in immature embryos and was cloned by *cis*-element interaction screening. HaDREB2 belongs to the DREB A2 subgroup of proteins that interact with drought-responsive element (DRE) sequences found in plant promoters [9,10]. DREB2 proteins are involved in the response and acclimation to dehydration and to high-temperature stress, whereas related DREB1 proteins are induced by cold. The DREB1 and DREB2 transcription factors and related AP2, ERF, and RAV1 groups belong to the APETALA2/ethylene-responsive element binding protein (AP2/ERBP) family, which contains a total of 147 members in Arabidopsis [9,11]. We showed that HaDREB2 interacted with DRE sequences in the *Hahsp17.6G1 *promoter and that this interaction was required for synergistic transcriptional activation of *Hahsp17.6G1 *by HaDREB2 and HaHSFA9 in sunflower embryos [10]. We also pointed out sequence similarities and differences between HaDREB2 and other DREB2 factors in Arabidopsis and other plants. These differences might indicate the functional divergence of HaDREB2 from AtDREB2A. Thus, the most overall similar factor in Arabidopsis, AtDREB2A, appeared to differ from HaDREB2 in sequences conserved in DREB2 factors such as CrORCA1, from *Catharanthus roseus*, a plant species that is closer to sunflower than Arabidopsis. Such sequences included the carboxyl terminal region, which is possibly involved in transcriptional activation [10].

Very few plant DREB2 factors have been functionally characterized. In Arabidopsis for example, overexpression of AtDREB2C enhanced basal thermotolerance in vegetative tissues without causing negative effects on plant growth [12]. Similarly, a wheat DREB2 factor improved freezing and osmotic stress tolerance in transgenic tobacco, although some lines showed delayed germination [13]. The constitutive or stress-inducible expression of the maize ZmDREB2A factor in Arabidopsis plants improved their drought stress tolerance and basal thermotolerance. In this case the transgenic plants showed delayed bolting and reduced growth of rosette leaves [14]. In contrast with the above results, the overexpression of AtDREB2A or rice OsDREB2A factor in Arabidopsis was not sufficient for observing stress-tolerant phenotypes, but nor did it impair plant growth or development [15,16]. These DREB2 proteins could be unstable and/or have little transcriptional activity. Both proteins could require post-translational modification(s) that stabilize and activate them in the nucleus. AtDREB2A has been the most functionally analyzed of the plant DREB2 factors. Recent work identified E3 ubiquitin ligases named DRIP1 and DRIP2, which interact with and mediate AtDREB2A ubiquitination. The overexpression of DRIP1 and DRIP2 delayed the drought-stress response that is regulated by AtDREB2A [17]. Internal deletion of amino acids 136 to 165 transformed AtDREB2A into AtDREB2A CA, a constitutively active and stabilized form in the nucleus. That form, when overexpressed induced significant tolerance to drought stress [18]. In addition, AtDREB2A CA induced not only drought-responsive genes but also heat-shock-related genes; basal thermotolerance was increased in plants overexpressing AtDREB2A CA and decreased in AtDREB2A knockout plants [19]. AtDREB2A was shown to be induced by heat stress, and in turn specifically induced transcription of AtHSFA3, one of the 21 different HSFs in Arabidopsis. The AtDREB2A-mediated AtHSFA3 induction regulates the expression of heat-shock related genes involved in the observed thermotolerant phenotypes [20]. Recent analyses of genetic responses involved in plant acclimation to high temperature pointed to an additional DREB2 factor. AtDREB2B, together with AtHSFA3, are the only two transcription factors among the genes that were specifically induced in thermotolerant lines of Arabidopsis. Moreover, it appears that AtDREB2A and AtDREB2B could have some functional redundancy in thermotolerance, as mutants for either factor did not show a defect in heat acclimation [21]; *dreb2a-1 *mutant Arabidopsis plants, however, showed reduced basal thermotolerance when directly treated at 46°C for 45 min [22].

Here we report the results of a functional analysis of HaDREB2 in transgenic tobacco and point to clear differences with other functionally characterized DREB2 transcription factors. We overexpressed HaDREB2 using cauliflower mosaic virus *35S *(*CaMV35S*) promoter and enhancer sequences. We could easily detect accumulation of the HaDREB2 protein in vegetative tissues; however, HaDREB2 did not induce heat-shock protein genes, as cytosolic sHSPs (CI and CII) or HSP101 or increase basal thermotolerance. We also overexpressed HaDREB2 under the seed-specific *DS10 *gene regulatory sequences [[23], see also [3]]. In this case, gain-of-function phenotypes were observed, but only after the conjoint overexpression in seeds of HaHSFA9 and HaDREB2. The phenotypes included enhancement of the accumulation of sHSPs (CI and CII), of basal thermotolerance, and increased resistance to artificial aging. The strict dependence on a seed-specific HSF of the effects of HaDREB2 characterize the latter as a distinct DREB2 factor with unique properties and functions that are restricted to seed development. The conjoint overexpression of HaHSFA9 and HaDREB2 in vegetative tissues did not further enhance the ectopic accumulation of seed sHSPs (CI and CII) that is induced by HaHSFA9. Tolerance to severe dehydration is not increased beyond that observed upon constitutive overexpression of HaHSFA9 alone [4]. We discuss how the novel observations reported here would: A. – allow the functional assignment of HaDREB2 and of similar plant DREB2 factors; B. – indicate additional complexity in the transcriptional control of the HSFA9 program (i.e., involvement of additional, seed-specific factors or modifications); C. – improve the genetic modifications of seed longevity based on HSFA9 overexpression.

## Results

### Constitutive overexpression of HaDREB2: lack of effect on vegetative thermotolerance

**Figure 1:**
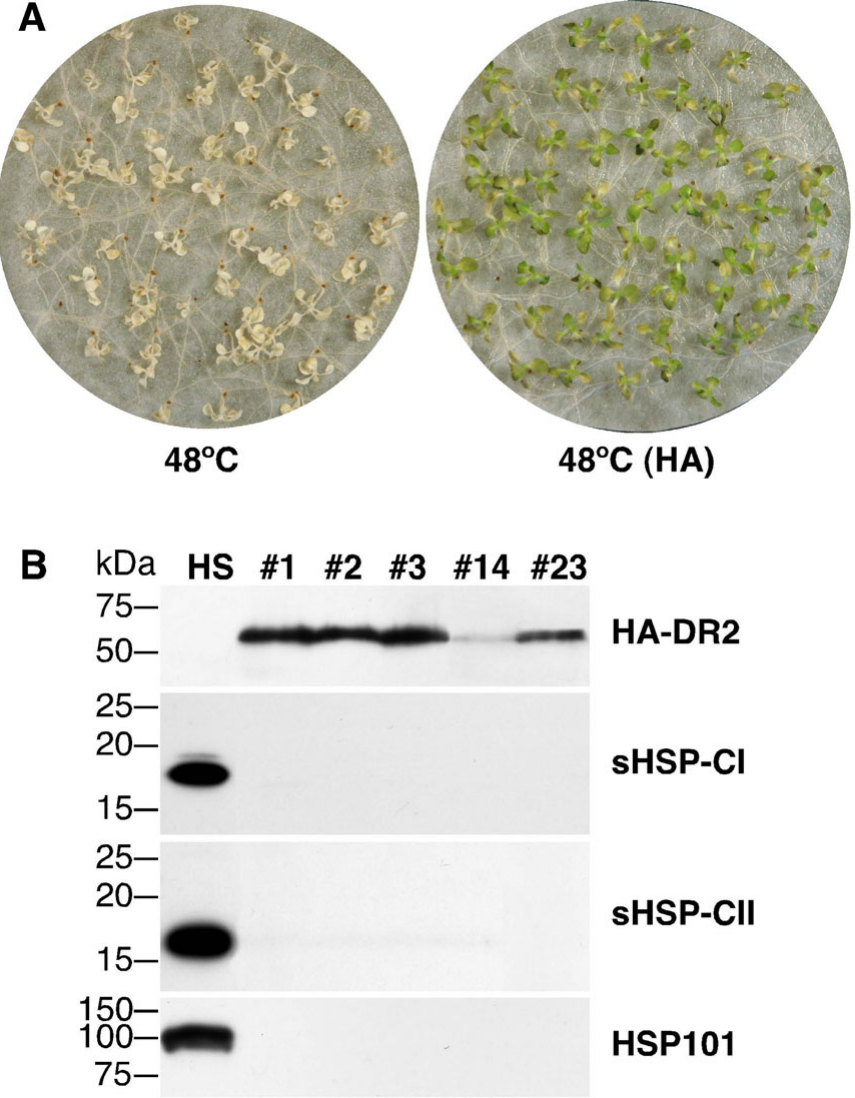
**Unaltered vegetative thermotolerance and HSP accumulation in heterozygous 35S:DR2 lines**. A. Seedlings from the 35S:DR2 lines do not survive direct exposure to 48°C for 2.5 h (48°C). However the 35S:DR2 seedlings survive the same heat stress treatment following heat acclimation [48°C (HA)]. Representative pictures of the 35S:DR2#1 line are shown here. B. Western blot showing accumulation of HA-tagged HaDREB2 (HA-DR2) in the 35S:DR2 lines. Detection with antibodies against the HA tag. The accumulation of different HSPs was detected in heat stressed, control plants (HS) but not in unstressed 35S:DR2 plants from the analyzed lines. The HSP-specific antibodies used for immunodetection are indicated on the right. Molecular mass markers (in kDa) are indicated on the left.

Based on previous results showing that at least some plant DREB2 proteins enhance vegetative thermotolerance [e.g. [12,14]], we tested the overexpression of HaDREB2 under *CaMV35S *sequences in transgenic tobacco, in 35S:DR2 plants. Basal thermotolerance and the accumulation of different HSPs at normal growth temperature (e.g. without heat stress) were analyzed in various 35S:DR2 lines. Each 35S:DR2 line carries a different, single integration of the *CaMV35S: HaDREB2 *transgene in heterozygosis. Representative results of these experiments are depicted in Figure 1. All seedlings from the 35S:DR2 lines died after exposure to a temperature of 48°C for 2.5 h. In contrast, seedlings from the same lines resisted the treatment at 48°C only if heat-acclimated by a previous treatment at a sub-lethal temperature (for 3 h at 40°C). Similar results were obtained with control, non-transgenic seedlings. In addition, 35S:A9 plants survived the 48°C treatment without previous heat acclimation (data not shown). This result, as previously reported [4], provided a positive control for partial, basal thermotolerance.

A hemaglutinin (HA) tag fused to HaDREB2 allowed us to determine whether protein stability is high enough for HaDREB2 to accumulate in transgenic tobacco. The tagged HaDREB2 could be readily detected to accumulate in the 35S:DR2 seedlings. In contrast, we could not detect accumulation of CI or CII sHSPs, nor of HSP101, in unstressed 35S:DR2 seedlings (Figure 1B). In summary, the HaDREB2 protein was stable enough to accumulate in transgenic tobacco seedlings; however, HaDREB2 overexpression did not modify basal or acquired thermotolerance or induce HSPs at normal growth temperatures. These findings contrast with previous results obtained with other DREB2 transcription factors [e.g. [12,14]].

### Seed-specific overexpression of HaDREB2: no effect on basal thermotolerance

**Figure 2:**
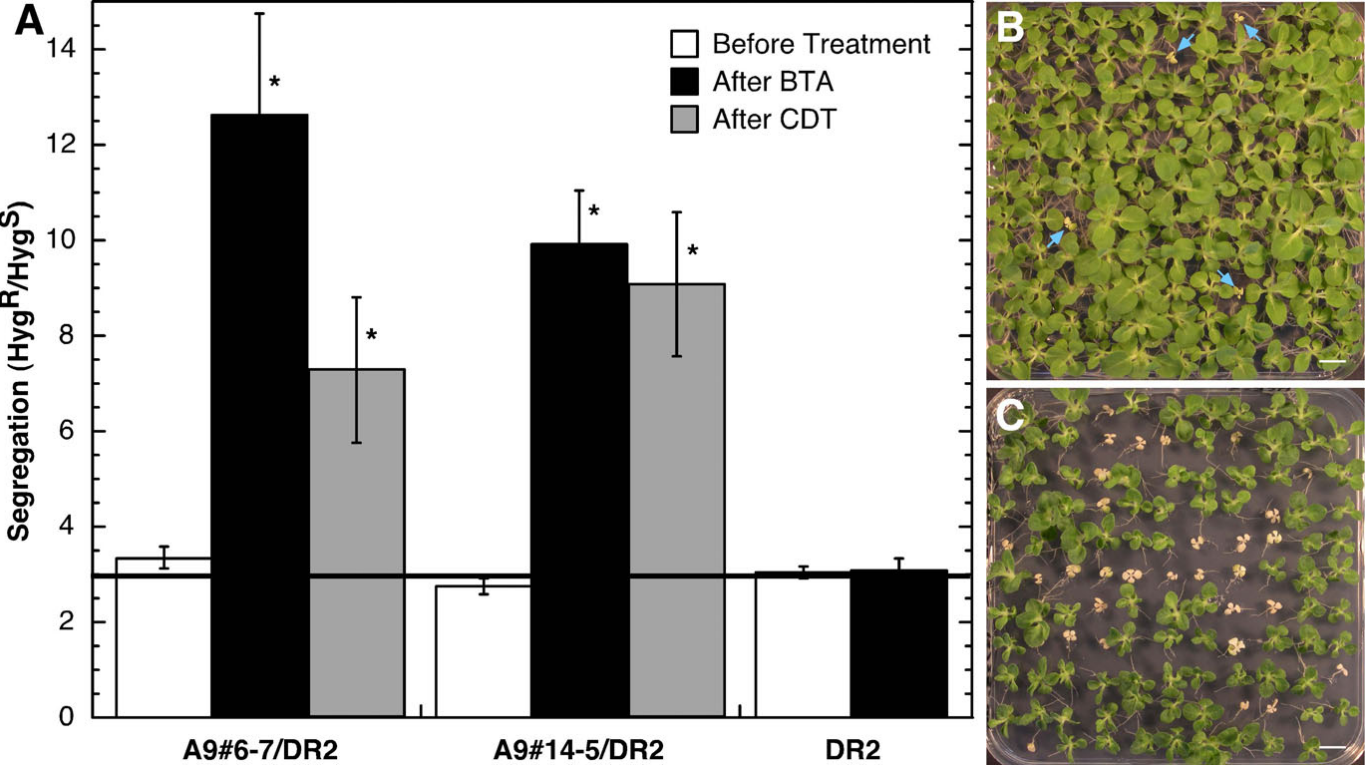
**Improved thermotolerance and enhanced resistance to CDT in seeds from the DS10:A9/DR2 heterozygous lines**. Segregation of hygromycin resistance (Hyg^R^) was analyzed in germinating seeds before and after the indicated treatment. BTA, basal thermotolerance assays; CDT, controlled deterioration treatments. Experiments were performed at least in triplicate for each line. A. – Combined results from five lines with the DS10:A9#6-7 homozygous background (A9#6-7/DR2), four lines with the DS10:A9#14-5 background (A9#14-5/DR2), and eight DS10:DR2 lines (DR2) are shown. Segregation was analyzed five (in DS10:A9/DR2 lines) or eight days (in DS10:DR2 lines) after BTA. Segregation was analyzed five days after CDT. The expected segregation ratio for antibiotic resistance is indicated with a thick line (marking 3 Hyg^R^: 1 Hyg^S^). Asterisks indicate differences that were found to be statistically significant (P < 0.05). We show representative pictures of segregation of hygromycin resistance for the DS10:A9#6-7/DR2#22 line after BTA (B) and before BTA (C). Arrows in (B) indicate the few Hyg^S ^seedlings that survived BTA. Scale bars, 8 mm.

We also tested, in DS10:DR2 plants, overexpression of HaDREB2 under the *DS10 *sequences previously used to confer the very efficient seed-specific expression of HaHSFA9 [3]. We analyzed various DS10:DR2 lines for persistence of basal thermotolerance after controlled seed imbibition. Basal thermotolerance assays (BTA) were thus performed [3] with seeds from different DS10:DR2 lines, each one with a single transgenic integration event in heterozygosis. No effects on basal thermotolerance were observed in eight DS10:DR2 lines studied. The *DS10:DR2 *transgene was linked to a marker for hygromycin resistance (Hyg^R^). Mendelian analyses were performed and the observed segregation ratios were evaluated by statistical analyses. Antibiotic resistance segregated at the expected 3:1 ratio both before and after the BTA (F = 0.01, P = 0.976, see Figure 2). This indicates that, after exposure for 4 h to 50°C, there was no difference in survival between segregating transgenic (homozygous or heterozygous for *DS10:DR2*) and sibling non-transgenic seeds. Only about 20% of the seeds survived BTA. Thus, basal thermotolerance in the DS10:DR2 seeds was lost after imbibition and exposure to 50°C. This result is similar to that previously observed for different non-transgenic and for some transgenic tobacco seeds without an enhanced thermotolerant phenotype, such as 35S:A9 [3]. The germination success of dry mature, DS10:DR2 seeds without stress treatment was not affected (95% to 100% in all lines). In agreement with the lack of effect of DS10:DR2 on thermotolerance, we observed similar accumulation levels of CI and CII sHSPs and of HSP101 in the seeds of DS10:DR2 lines compared to non-transgenic seeds (Additional File 1).

### Combined overexpression of HaDREB2 and HaHSFA9: further enhancement of thermotolerance and longevity in seeds

The failure to observe an effect of HaDREB2 on sHSP accumulation and thermotolerance in seeds (Figure 2, Additional File 1) could have alternative explanations. In this way, the overexpression of HaDREB2 alone (in the DS10:DR2 lines) would be insufficient if the expression levels of endogenous HSFA9 factor were insufficient and/or if the properties of HSFA9 factors from tobacco and sunflower were different. For example, the tobacco HSFA9 factor and HaHSFA9 could be optimized in each case for functional interaction with a homologous DREB2 factor, HaDREB2 in sunflower. Our previous work lends support to the fact that the level of tobacco HSFA9 is insufficient. HaHSFA9 and tobacco HSFA9 did not differ in the seed regulation of wild type (WT) and mutant (in heat shock *cis*-elements) versions of the *Hahsp17.7G4 *promoter [1]; this observation supported our decision to use tobacco as a suitable heterologous system (with similarly evolved HSFA9 and *sHSP *target genes). To deal with any limiting factor in the heterologous system, we combined overexpression of HaDREB2 and HaHSFA9 in seeds of transgenic tobacco. DS10:A9/DR2 lines were obtained by transforming homozygous DS10:A9 lines, which have been described to show enhanced and stable thermotolerant seed phenotypes. Seeds from these parental lines also showed resistance to controlled-deterioration, a procedure for rapid aging [3]. The DS10:A9/DR2 lines were produced from two different DS10:A9 backgrounds, these being the previously characterized transgenic lines DS10:A9#6-7 and DS10:A9#14-5 [3].

Initial experiments were performed with DS10:A9/DR2 lines that contained different, single, integration sites of the *DS10:HaDREB2 *transgene in heterozygosis. This was combined with a single integration of the *DS10:HaHSFA9 *transgene in homozygosis (a different integration event in each parental background). These lines were analyzed for persistence of basal thermotolerance by analyzing Mendelian segregation of the Hyg^R ^marker gene among seeds that survived exposure to high temperature in BTA. Because the DS10:A9 parental lines already showed enhanced resistance in BTA [3], exposure to 50°C was prolonged to 5 h. Seeds that combined the *DS10:HaHSFA9 *(linked to kanamycin resistance) and *DS10:HaDREB2 *(linked to Hyg^R^) transgenes showed enhanced persistence of basal thermotolerance. This was strongly indicated by a clear increase after BTA of Hyg^R ^ratios among seeds that survived the treatment. Thus, ratios of Hyg^R ^to Hyg^S ^increased from the expected 3:1 value to values up to 17:1. This increase was consistently observed in independent experimental repetitions performed with nine lines derived from two different parental lines. Statistical analyses confirmed a highly significant effect on segregation after BTA for the *HaDREB2 *gene, when combined with *HaHSFA9 *in either parental background (for A9#6-7, F = 93.97, P = 0.001; for A9#14-5, F = 116.18, P = 0.001). The results of these experiments are summarized in Figure 2, allowing for direct comparison with those for DS10:DR2 lines that were described above (the lack of effect of the single overexpression of HaDREB2 in seeds). It is clear that a positive effect of HaDREB2 overexpression on seed thermotolerance requires the concurrent overexpression of HaHSFA9.

In our previous report on seed-specific overexpression of HaHSFA9 [3], we showed that the BTA results agree with those of standard procedures used for rapid seed aging and for assessment of seed longevity. One such procedure is controlled deterioration treatment (CDT) [3,24,25]. Recent work has demonstrated that CDT and natural seed aging involve similar molecular events, such as oxidation of identical target proteins in Arabidopsis [26]. These findings facilitate the analysis of seed longevity in Solanaceae plants, which already resist aging much more on average than that seen in other plants, even without genetic modification [3,27]. Natural-aging experiments involving wild type tobacco seeds could thus take several years to complete, e.g., by testing storability at room temperature. Therefore, the effect of HaDREB2 on seed longevity that is indicated by the BTA results in Figure 2 was confirmed using the CDT procedures that we described for tobacco seeds [3]. The results included in Figure 2 also show that HaDREB2 and HaHSFA9 enhanced resistance to CDT for 2 d at 50°C (e. g. enhanced seed longevity) in a similar way to that indicated by the BTA results. Thus, statistical analyses also confirmed a highly significant effect on segregation after CDT for the *HaDREB2 *gene in both parental *HaHSFA9 *backgrounds used (for A9#6-7/DR2, F = 7.28, P = 0.017; for A9#14-5/DR2, F = 15.01, P = 0.0017).

Further to the positive effect on thermotolerance and longevity, we also observed hints of a deleterious effect of HaDREB2 overexpression in seeds. In this way, the DS10:A9/DR2 plants produced normal seeds and in normal yield, but the germination percentage of unstressed, mature seeds was reduced from 95% to 40% in 10 out of 63 lines with the *DS10:DR2 *transgene in heterozygosis. However, in most lines showing defective germination, segregation was consistent with the multiple integration of *DS10:DR2*. This contrasted with the mentioned lack of effects of single overexpression of HaDREB2 in the DS10:DR2 seeds. Therefore, both the positive and negative effects of HaDREB2 required concurrent overexpression of HaDREB2 and HaHSFA9 (Figure 2). However, in a high proportion (≅5/6) of the DS10:A9/DR2 lines, seeds showed unaltered germination under unstressed conditions. These lines and their progeny were thus used for the thermotolerance and CDT experiments in Figure 2 and for subsequent analyses (see below).

**Figure 3:**
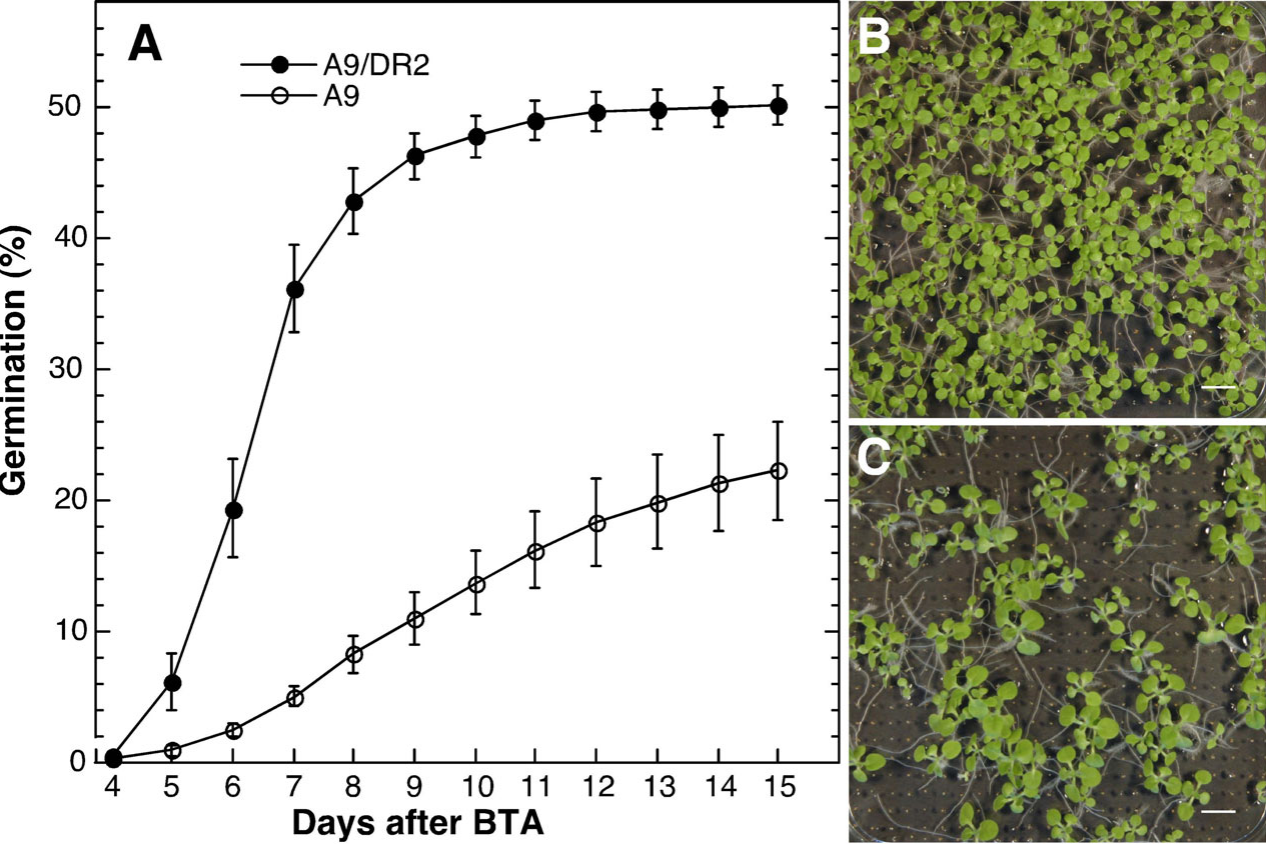
**The combined *DS10:DR2 *and *DS10:A9 *transgenes in homozygosis enhance seed survival after BTA**. A. Germination after BTA of double-homozygous seeds (A9/DR2) compared to that of seeds from sibling lines without DR2 (A9). We show average germination at different time after BTA for 3 to 4 independent experiments performed with two pairs of A9/DR2 lines (A9#14-5/DR2#5-7 and A9#14-5/DR2#23-5) compared to their respective sibling lines without DR2 (A9#14-5/#5-4 and A9#14-5/#23-6). Also shown are pictures of representative results of individual experimental samples showing germination 15 d after BTA: B. A9#14-5/DR2#5-7 (A9+DR2). C. A9#14-5/#5-4 (A9). Scale bars, 10 mm.

The effects of the *DS10:DR2 *transgene were confirmed in second-generation seeds. From three representative DS10:A9/DR2 lines (heterozygous for *DS10:DR2 *in the two *DS10:A9*, homozygous, parental backgrounds), we obtained after segregation the respective pairs of sibling lines with the *DS10:DR2 *transgene in homozygosis (DS10:A9#6-7/DR2#25-2; DS10:A9#14-5/DR2#23-5; DS10:A9#14-5/DR2#5-7), and without the *DR2 *transgene (DS10:A9#6-7/#25-1; DS10:A9#14-5/#23-6; DS10:A9#14-5/#5-4); all of these lines also had the *DS10:A9 *transgene in homozygosis. Performing BTA enabled assessment of the enhanced persistence of thermotolerance. Here (see Figure 3), we used a higher challenging temperature and for a shorter time (treatment at 52°C for 4 h) compared to conditions used for assays with the heterozygous parental lines (see Figure 2). The results summarized in Figure 3 demonstrate that the *DS10:DR2 *transgene in homozygosis enhanced seed survival after BTA compared to sibling lines with only *DS10:A9*. Seeds that did not geminate after BTA were dead, as previously observed for the parental *DS10:A9 *lines [3]. Comparisons are separated for the A9#14-5 (Figure 3) and A9#6-7 (Additional File 2) parental genetic backgrounds, thus better controlling initial differences in thermotolerance (e.g. caused by differences in HaHSFA9 overexpression). In addition, within each comparison, we used sibling lines differing only in the inheritance and expression of the *DS10:DR2 *transgene (and of downstream genes). This provides a necessary control for epigenetic variability [3,4]; e.g., somaclonal variation, which is a problem that is inherent to tobacco transformation and regeneration *in vitro*. We confirmed that the additional effect of HaDREB2 on survival was statistically significant in both the A9#6-7 and A9#14-5 backgrounds. For example, differences between the germination percentages of single and double-homozygous seeds were highly significant over the duration of the experiments in Figure 3 (4–15 d after BTA [F = 102.54, P = 0.0001, 1 and 121 df; repeated-measures ANOVA]). Figure 3(B–C) shows pictures from a representative experiment performed with one of the sibling pairs. Besides the obvious difference in percent germination after BTA, sibling DS10:A9/DR2 and DS10:A9 seedling growth was similar; no difference in seedling size was apparent 15 d after BTA (Figure 3B–C). This indicates that HaDREB2 did not improve seedling growth after BTA further than it was previously observed with the single overexpression of HaHSFA9 [3].

### The combined overexpression of HaDREB2 and HaHSFA9 induces subtle changes in the accumulation patterns of seed sHSPs

**Figure 4:**
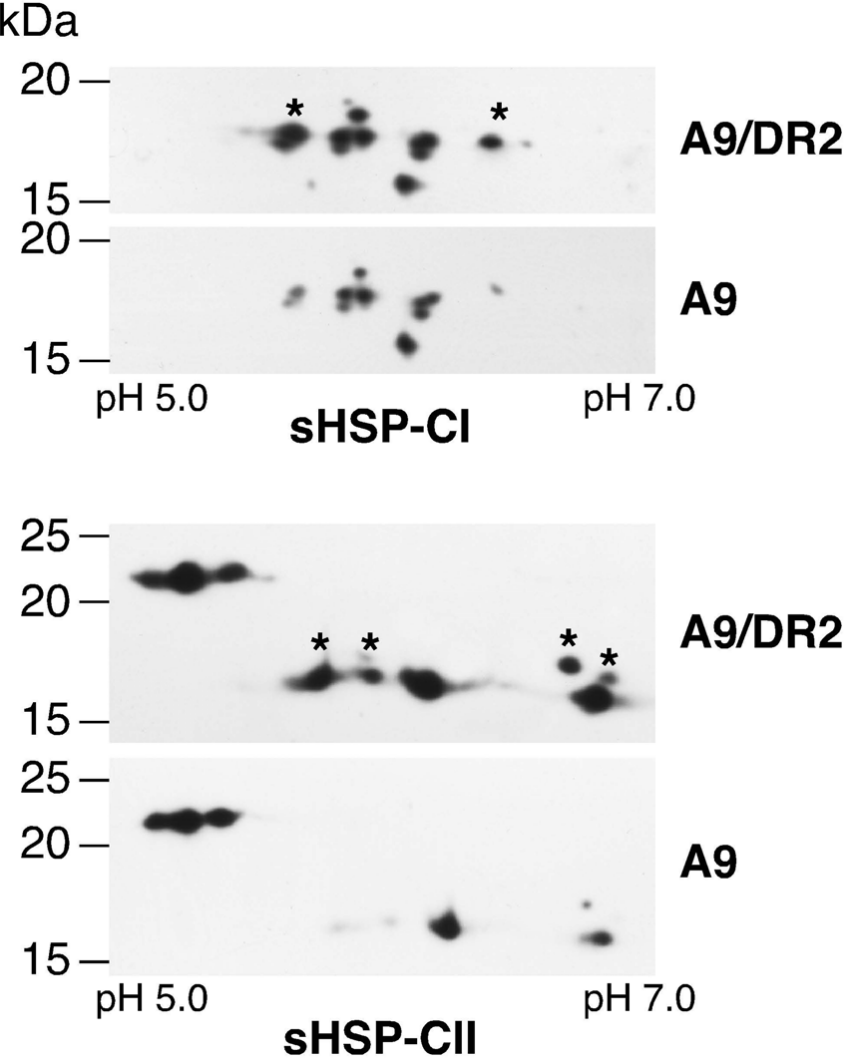
**Specific enhancement of the accumulation of some sHSPs in seeds of DS10:A9/DR2 lines**. Comparison of the accumulation patterns of sHSP-CI and sHSP-CII. A representative, double-homozygous DS10:A9/DR2 line (A9/DR2) is compared to its sibling DS10:A9 line (A9). Asterisks mark the polypeptides that consistently showed higher accumulation in the DS10:A9/DR2 lines. Molecular mass markers (in kDa) are indicated on the left. The pH range for isoelectric focusing (IEF) is indicated (bottom).

We also analyzed HSP and dehydrin protein accumulation in seeds from the same lines that were used for the BTA experiments outlined in Figure 3. The use of "syngenic" sibling seed-material was essential for the detection of subtle changes that could be otherwise hidden by epigenetic variation. Indeed, western analyses performed after 1D-electrophoresis only revealed a very slight, almost imperceptible, increase in the accumulation of CI sHSPs in seeds of the DS10:A9/DR2 lines. A slightly more pronounced increase was seen for CII sHSPs, and it appeared clearer for a minor protein species of 19.7 kDa that reacted with antibodies against plant dehydrins (Additional File 3). The accumulation enhancement of CI and CII sHSPs was confirmed by western analyses performed after 2D-electrophoresis. The results depicted in Figure 4 show that HaDREB2 specifically enhanced the accumulation of some of the seed CI and CII sHSPs. In the case of CI sHSPs, the affected polypeptides included a triplet of acidic spots that are resolved within the major 18 kDa band. The most basic polypeptide of the same size class was also induced by HaDREB2 (Figure 4, top). This corresponds to a minor spot for a heat-inducible polypeptide that is barely detectable in DS10:A9 and non-transgenic seeds [3]. Regarding the CII sHSPs, we observed an enhancement of the accumulation of all spots resolved around a size of 15 kDa. Such spots correspond to seed polypeptides that are also induced by heat stress [3]. This result contrasted with the lack of effect of HaDREB2 on the accumulation of seed-specific CII sHSPs, which correspond to the spots resolved around an average size of 22 kDa (Figure 4, bottom). All the changes mentioned here were consistently observed in independent experimental repeats performed with the three pairs of sibling lines.

The content of total soluble carbohydrate was analyzed in seeds from two sibling pairs of lines representing each parental genetic background. The results in Additional File 4 show that total soluble carbohydrate was the same for DS10:A9 and DS10:A9/DR2 seeds. This result was not unexpected given that the single overexpression of HaHSFA9 did not affect the accumulation of total soluble carbohydrate in seeds, in contrast with the reported effect on seed HSPs [3].

### The combined overexpression of HaDREB2 and HaHSFA9 does not confer further dehydration tolerance in seedlings

**Figure 5:**
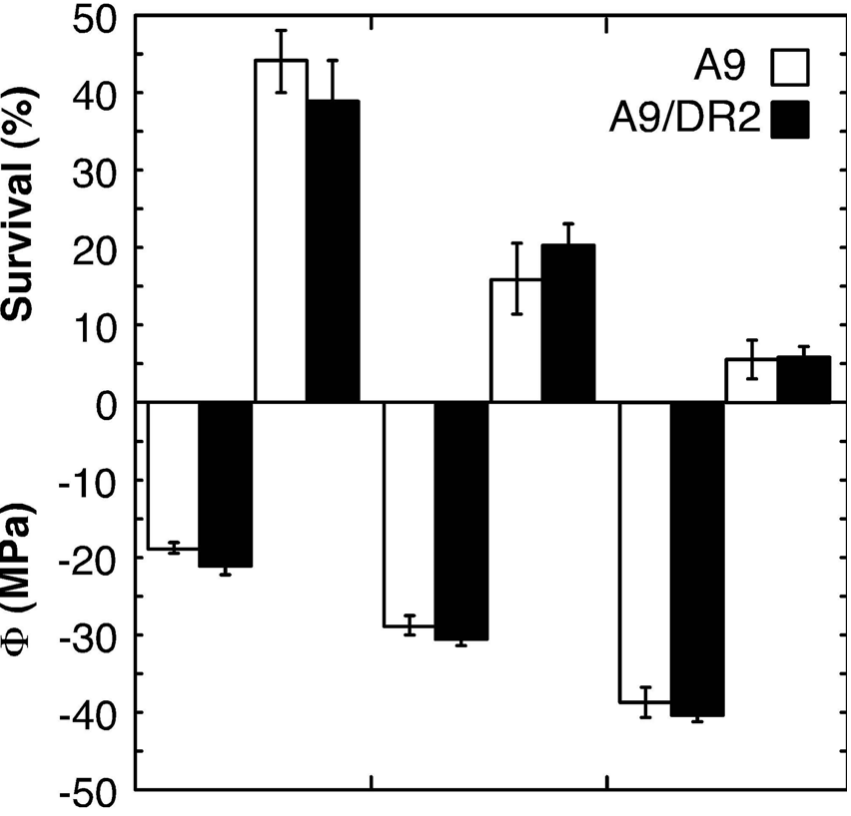
**The *35S:DR2 *transgene when combined with *35S:A9 *does not further enhance tolerance to severe dehydration**. Average survival of whole seedlings after DT2 assays (top). Average water potential (Φ) reached in the different DT2 assays (bottom). Data were obtained from two 35S:A9/DR2 lines (A9/DR2), 35S:A9#2-18/DR2#1-6, 35S:A9#12-3/DR2#22-3, and the corresponding sibling 35S:A9 lines (A9). Each pair of sibling lines represents the genetic background of the previously analyzed parental 35S:A9 lines [4]. Data were obtained from 3 to 14 experimental repeats per line and average water potential condition.

The results in Figures 2, 3 and 4 and Additional File 2 demonstrate a functional interaction between HaDREB2 and HaHSFA9 in seeds of transgenic tobacco. To investigate if a similar interaction occurs in vegetative tissues, we combined the overexpression of the two transcription factors under *CaMV35S *sequences. Double-homozygous 35S:A9/DR2 lines, and their respective 35S:A9 siblings, were obtained from two different parental backgrounds (the previously characterized 35S:A9#2-18 and 35S:A9#12-3 lines), which carry a single integration of the *35S:A9 *transgene in homozygosis [4]. The *35S:DR2 *transgene when combined with *35S:A9 *did not further enhance the tolerance to severe dehydration caused by the single overexpression of HaHSFA9. This was determined by DT2 experiments [4] in which whole seedlings from double-homozygous 35S:A9/DR2 lines survived dehydration to the same extent as the respective "syngenic" 35S:A9 material. Figure 5 summarizes the results of these experiments, performed with two pairs of sibling lines, with each pair representing the mentioned parental backgrounds. Additional experiments showed that the survival of green organs was also not affected (Additional File 5). We could not detect an enhancement in the ectopic accumulation of sHSPs (see Additional File 5) that is caused by overexpression of HaHSFA9 [4]. We also failed to detect any effects of the *35S:DR2 *transgene on the basal thermotolerance of seedlings that is induced by overexpression of HaHSFA9 [4], or on acquired thermotolerance (data not shown). Thus, 35S:A9/DR2 and sibling 35S:A9 seedlings could heat-acclimate in a similar way, as was previously described for non-transgenic tobacco and for 35S:A9 material [4]. Therefore, HaDREB2 does not appear to interact with the HSFs involved in vegetative heat-acclimation in tobacco. Furthermore, an interaction between HaDREB2 and HaHSFA9 could not be observed in vegetative organs of seedlings.

## Discussion

The results presented here demonstrate a functional interaction between the transcription factors HaDREB2 and HaHSFA9 (from sunflower) in transgenic tobacco. The conjoint seed-specific overexpression of both factors was required in order to observe enhanced phenotypes related to seed longevity, as demonstrated by improved resistance to CDT [3,26]. The observed phenotypes occurred in concurrence with subtle and specific increases in the accumulation of seed sHSPs. However, in seedlings we could not detect effects of HaDREB2 on HSPs, thermotolerance, or dehydration tolerance, beyond what is observed by overexpressing only HaHSFA9. Our results strongly indicate unique properties for HaDREB2 in connection with HSFs, seed development and seed longevity. This would set apart HaDREB2 from previously characterized DREB2 factors in plants.

We found that HaDREB2 differs from other characterized DREB2 factors in terms of its stability and its functional interaction with HaHSFA9, a class A HSF transcription factor with expression patterns and roles mostly restricted to seeds [1]. Thus, we could easily detect the accumulation of the HA-tagged HaDREB2 protein in both the 35S:DR2 and DS10:DR2 lines (Figures 1B and Additional File 1). This contrasts with previous reports on AtDREB2A, a DREB2 protein from Arabidopsis. The AtDREB2A protein could be detected in vegetative tissues, but only upon deletion of amino acids 136 to 165 [18]. The observed stability of the HaDREB2 protein in vegetative tissues is consistent with the absence, in HaDREB2, of similar sequences as in the domain involved in the instability of other DREB2 proteins such as AtDREB2A or rice OsDREB2A [[16,18], Additional File 6].

HaDREB2 is also unlike other DREB2 factors such as AtDREB2C, ZmDREB2A, and WDREB2 from Arabidopsis, maize and wheat, respectively. These proteins would also differ from AtDREB2A in their stability in vegetative tissues. Thus, the respective intact proteins have been found to be active when overexpressed in homologous, or even heterologous transgenic plants. AtDREB2C, ZmDREB2A and WDREB2 were able to enhance resistance to heat [12,14], cold [13], or osmotic stress [12,13]. The abiotic stress resistance was observed without additional negative effects for AtDREB2C only [12], whereas the ZmDREB2A transgenic plants showed delayed bolting and reduced growth [14], and some WDREB2 lines showed delayed germination [13]. In contrast, and despite the observed accumulation of the protein in vegetative tissues, HaDREB2 did not enhance thermotolerance (Figure 1) or dehydration tolerance (Figure 5 and Additional file 5). Furthermore, overexpression of HaDREB2 in vegetative tissues did not negatively affect growth or development.

The sensitivity to heat stress of the 35S:DR2 lines shows that HaDREB2 did not activate the genetic programs associated with thermotolerance that involve different DREB2 factors. These programs involve transcriptional activation of several *HSP *genes [i.e., [19-21]], whereas the 35S:DR2 lines did not show altered expression of HSP101 or of cytosolic sHSPs (CI, CII). Our results suggest that HaDREB2 does not activate transcription factors associated with vegetative thermotolerance. Among these are HSFs that could differ between different plant species. For example, only AtHSFA3, one of the 21 different Class A HSFs encoded by the Arabidopsis genome, was found to be a potential, direct target of AtDREB2A [20,22]. Different Class A HSFs such as AtHSFA1a, AtHSFA1b, AtHSFA1e, AtHSFA3, and AtHSFA7 have been reported to contribute to vegetative thermotolerance and to *HSP *gene transcriptional activation in Arabidopsis [19-21,28]. In contrast, in tomato plants a single HSF, LpHSFA1, appears to play a master role in vegetative thermotolerance [29]. Whatever the HSF(s) involved in tobacco (which, like tomato, is a Solanaceae), HaDREB2 would fail to induce them, or to interact functionally with them on *HSP *gene promoters. This interpretation fits with the lack of effect on vegetative thermotolerance and HSP expression in the 35S:DR2 lines.

On the other hand, the combined effects of HaDREB2 and HaHSFA9 on thermotolerance, CDT resistance, and HSP expression in transgenic tobacco seeds would confirm, in a heterologous system, a functional interaction between both factors. We previously reported in sunflower embryos evidence for such an interaction, which was observed on a single promoter belonging to the *CI sHSP *gene family. The HSFA9-specificity involved in the reported interaction [10] and the seed-specific expression patterns of HSFA9 proteins [1,2] would agree with the observed enhancement of thermotolerance in tobacco seeds, and the lack of functional effects in the 35S:DR2 lines. The specific functional interaction between HaDREB2 and HaHSFA9 would additionally set HaDREB2 apart from other characterized DREB2 plant proteins. Based on the effects observed in the DS10:A9/DR2 lines, we conclude that HaDREB2 contributes to the genetic program of seed-longevity and embryo desiccation tolerance regulated by HaHSFA9. We previously suggested that the specificity of the synergistic interaction between HaDREB2 and HaHSFA9 could involve unique sequences, which in HaHSFA9 included its carboxyl-terminal activation domain [10]. We also would like to point out other sequences that are located in the putative carboxyl-terminal activation domain of HaDREB2. Such sequences may be conserved in some DREB2 proteins such as DvDREB2A (the protein most similar to HaDREB2 [30]), but not in AtDREB2A (see [10] and Additional File 6).

HaDREB2 and HaHSFA9 only interact inefficiently in GST-pull down assays [10]. However, physical interaction between HaDREB2 and HaHSFA9 is not very likely to occur *in planta *as conditions that would facilitate physical interaction are detrimental for the transcriptional synergism between these two factors [10]. This synergism requires the independent binding of both factors to different *cis*-elements in the *Hahsp17.6G1 *promoter [10]. We hypothesized that the same mechanism could be used for transcriptional activation of other *sHSP *promoters [10]. Our observations in transgenic tobacco seeds are consistent with such a proposal. Only a subset of the proteins encoded by the tobacco CI *sHSP *genes increased their accumulation in the DS10:A9/DR2 lines (Figure 4). In contrast, all CI sHSPs that are detected in tobacco seeds increased their accumulation in the DS10:A9 lines [3]. A similar specificity was evident by comparing the levels of CII sHSPs in seeds of the DS10:A9/DR2 (see also Figure 4) and DS10:A9 lines [3]. The specific effects of HaDREB2 on seed-sHSP accumulation would indicate that HaDREB2 coactivates (with HaHSFA9) only a subset of the gene promoters activated by HaHSFA9 alone. For *sHSP *promoter activation HaDREB2 needs a functional DRE similar to one that we characterized in the *Hahsp17.6G1 *promoter [10]. Other promoters for *sHSP *genes expressed in plant seeds are yet to be functionally analyzed. Only some of the *sHSP *genes in fully sequenced genomes, such as Arabidopsis, show potential core DRE *cis*-elements within 1000 bp upstream of start codons (data not shown). However, functional DREs are very difficult to predict based only on DNA sequence information. The nucleotide sequence near the core DRE element is important [31,32], but the conclusive determination of that sequence context would require functional analyses of a number of different promoters. Lacking additional examples for *sHSP *promoters activated by DREB factors in plant seeds, we can only speculate on the involved sequence context. Its determination awaits the functional characterization of additional *sHSP *gene promoters known to be active in seeds. Whatever that context may be, we propose that it is different from that in other promoters induced in vegetative tissues by DREB proteins such as AtDREB2A. This could explain why no Arabidopsis CI or CII *sHSP *genes were predicted as target gene candidates for the former DREB2 proteins, as predictions were based on DRE sequence contexts determined for AtDREB2A and its target genes (see [32] and references therein). Thus, if DREB2 factors different from AtDREB2A are involved in the regulation of *sHSP *genes in Arabidopsis seeds, they could recognize a different DRE promoter context. HaDREB2 and other similar DREB2 proteins could recognize the DRE sequence context found in the *Hahsp17.6G1 *promoter, which would not be necessarily conserved in Arabidopsis *sHSP *genes. This proposal is consistent with the observation in the AP2 (DNA-binding) domain of HaDREB2 of amino-acid residues that are conserved in DvDREB2A, LeDREB1, and CrORCA1, but not in AtDREB2A (Additional File 6).

In seedlings of the 35S:A9/DR2 lines we could not detect effects of HaDREB2 on thermotolerance or dehydration tolerance beyond what is observed by overexpressing only HaHSFA9. This contrasts with the conjoint activity of HaDREB2 and HaHSFA9 in seeds of transgenic tobacco. In consequence, we propose that additional seed-specific regulators might be involved in the genetic program(s) controlled by HaHSFA9 [3,4] and HaDREB2 (this work). Vegetative organs could thus lack factors (or factor modifications) that would be necessary for the functional interaction between HaDREB2 and HaHSFA9. Such factors would be conserved in the heterologous system (transgenic tobacco) but present in sufficient amounts only in seeds. We will attempt to clone the hypothetical additional factors. Future work with these factors might confirm our proposal. Finally, we would like to point out that HaDREB2 could be used in combination with HaHSFA9 as a tool for genetic improvement of seed longevity, along the lines previously proposed for HaHSFA9 [3]. Transgenes expressing HaDREB2 and HaHSFA9 could eventually be stacked in elite hybrid cultivars [33] for enhanced effect on longevity.

## Conclusion

We conclude that HaDREB2 contributes to the genetic program of seed-longevity that is regulated by HaHSFA9. We demonstrated a functional interdependency of the transcription factors HaDREB2 and HaHSFA9 in seeds of transgenic tobacco. HaDREB2 would thus differ from other previously characterized DREB2 factors in plants in its unique functional interaction with the seed-specific HaHSFA9 factor. We pointed out amino-acid residues that are conserved in HaDREB2, DvDREB2A, LeDREB1, and CrORCA1, but not in AtDREB2A. The unique sequences in HaDREB2 and similar DREB2 proteins are located in DNA-binding and transcriptional activation domains, which would be consistent with their functional specialization. Distinct from AtDREB2A and similar DREB2 factors, the HaDREB2 protein appears to be stable enough to accumulate in vegetative tissues. Nevertheless, no functional interaction between HaDREB2 and HaHSFA9 was observed when both factors were conjointly overexpressed in vegetative tissues. We therefore suggest that additional, seed-specific factors, or protein modifications could be required for the functional interaction between HaDREB2 and HaHSFA9.

## Methods

### Plant materials

Tobacco (*N. tabacum *L. var. Xanthi) was used for all experiments. Seed sterilization, germination, and seedling growth under controlled conditions were as previously described in detail [4]. All experiments involving young plants were performed with 3-week old seedlings.

### Overexpression of HaDREB2 in transgenic plants

As parental lines for transformation with *HaDREB2 *transgenes (see below), we used WT *N. tabacum *L. (var. Xanthi) and previously characterized transgenic lines. These lines, DS10:A9 and 35S:A9, overexpress HaHSFA9 from seed-specific or constitutive promoters, respectively [3,4]. We have described previously in depth the procedures for tobacco transformation, and the protocols for selection of heterozygous transgenic lines with single transgene integration events [3,4]. Homozygous and sibling lines without each *HaDREB2 *transgene were obtained in the subsequent generation (after transgene segregation) [3,4]. Transgenic plants for genes that overexpress HaDREB2 were selected on medium with hygromycin (45 μg ml^-1^).

For constitutive overexpression of HaDREB2 in transgenic plants (35S:DR2 and 35S:A9/DR2 lines), the HaDREB2 coding sequences were PCR-amplified from plasmid pBluescript SK-HaDREB2 [10]. An *Nco*I restriction site was engineered immediately next to the start codon of HaDREB2, allowing in-frame fusion after the C-terminus of hemaglutinin-tag (HA) sequences. The fusion was cloned between the CaMV 35S (35S) promoter-enhancer and *nos *terminator sequences from plasmid pBHA . In brief, a *35S:HA:HaDREB2:nos *chimeric gene was first assembled in vector pUC19, yielding the pUC19-35S:HA:HaDREB2:nos plasmid. The nucleotide sequence of the HA:HaDREB2 fusion was verified by sequencing. A 1799 bp *Hind*III – *Sal*I fragment from the pUC19-35S:HA:HaDREB2:nos plasmid that contained the *35S:HA:HaDREB2:nos *gene was inserted in the binary vector pBIB-Hyg [34], and used for plant transformation.

For seed specific overexpression of HaDREB2 in transgenic plants (DS10:DR2 and DS10:A9/DR2 lines), the HA:HaDREB2 fusion was PCR-amplified, as a 1127 bp DNA fragment, from plasmid pUC19-35S:HA:HaDREB2:nos. Then it was cloned in the *Eco*RI site of vector pSK-ds10EC1 [35], after Klenow polymerase fill-in treatment of *Eco*RI ends. Thus, we obtained plasmid pSK-ds10EC1-HA:HaDREB2, in which the HA:HaDREB2 fusion is placed under *DS10 *promoter-enhancer sequences and followed by *DS10 *terminator sequences [35]. The primers used for PCR amplification were: 5'-TCTAGTAAAAATGGCACC-3' (HA-ATG) and 5'-CAAGATTCTACTTCTAGT-3' (HaDR2-3'A). The amplification mixture was first incubated for 1 min at 94°C. Thereafter, PCR was performed using *Pwo*-DNA polymerase (Roche) and the amplification conditions were as follows: 30 cycles of 30 s at 94°C, 30 s at 48°C, and 1 min at 72°C, plus a final step at 72°C for 5 min. The nucleotide sequence of the PCR-amplified DNA fragment was fully verified before the final cloning step. A DS10:HA:HaDREB2:DS10 DNA fragment of 4751 bp was obtained after *Sal*I and *Xba*I digestion of plasmid pSK-ds10EC1-HA:HaDREB2, and it was inserted in the corresponding restriction sites in the polylinker of the binary vector pBIB-Hyg [34].

### Seedling thermotolerance assays

Seedlings were exposed to a temperature of 48°C without a heat-conditioning treatment (in basal thermotolerance assays), or after a heat-conditioning treatment of 3 h at the sub-lethal temperature of 40°C (in acquired thermotolerance assays). The heat stress treatments were performed by immersion of sealed Petri dishes in water baths at each temperature. Upon completing treatments, seedlings were returned to normal growth conditions and were photographed 1 week after [4].

### Basal thermotolerance assays of seeds and controlled deterioration

We have described in depth [3] the conditions used for assaying the persistence of basal thermotolerance in tobacco seeds (BTA), as well as for their controlled deterioration (CDT). The specific temperature and treatment duration conditions employed for optimal evaluation of the DS10:DR2 and DS10:A9/DR2 lines were empirically determined. Thus, for the experiments in Figure 2 the temperature and duration of the treatment was chosen so that after treatment a sufficient number of seeds germinate on non-selective medium to allow statistical analyses of Hyg R/S segregation. BTA treatments at 50°C were for 5 h in the case of DS10:A9/DR2 lines and for 4 h in the DS10:DR2 lines. For the experiments in Figure 3 (and Additional File 2), we used more stringent BTA conditions (4 h, 52°C) than in Prieto-Dapena *et al.*, (2006). This allowed us to clearly detect the additive effect of DR2 in the DS10:A9/DR2 lines. Conditions used in each case are indicated in Results.

### Dehydration tolerance assays

DT2 assays with seedlings were performed following described procedures; the effects of severe dehydration on survival of leaves or whole seedlings were evaluated as described previously [4].

### Protein and soluble carbohydrate analysis

Protein electrophoresis (1D and 2D), Western-blot assays, and the analysis of soluble carbohydrates were performed as described previously [3,4]. The HA-tagged DREB2 protein was detected using anti-HA-peroxidase antibodies (high affinity 3F10, *Roche*) at 1/2000 dilution. HSP101 was detected with anti-Hsp101/ClpB N-terminal antibodies (*Agrisera*) at 1/20000 dilution.

### Statistical analyses

Differences between the transgenic and control groups of sibling seeds (or seedlings) were tested using ANOVA. For comparisons involving temporal responses (e.g. germination after the BTA or CDT assays), we used repeated-measures ANOVA. Statistical treatment has been previously described in depth [3,4].

## Authors' contributions

JDM and JME constructed the DR2 chimeric genes, transformed 35S:DR2 and DS10:DR2 into WT tobacco, and contributed to initial characterization of the 35S:DR2 lines. CA obtained the double transgenic lines (35S:A9/DR2 and DS10:A9/DR2) and sibling control lines. CA and PPD characterized the phenotypes of the double transgenic and sibling control lines. RC performed the 2D protein analyses of DS10:A9/DR2 and sibling control lines. PPD performed the statistical analyses. JJ conceived and coordinated the study and wrote the manuscript. PPD and CA edited the manuscript. All the authors read and approved the final manuscript.

## Supplementary Material

Additional file 1 **Accumulation of HSPs in seeds from DS10:DR2 lines to similar levels as in non-transgenic seeds**. Western blot analyses of HSPs in seeds from DS10:DR2 lines showing that their accumulation levels are indistinguishable from negative controls. Click here for file

Additional file 2 **Combined *DS10:DR2 *and *DS10:A9 *transgenes in homozygosis enhance seed survival after BTA: results in the DS10:A9#6-7 genetic background**. BTA assays showing that combined *DS10:DR2 *and *DS10:A9 *transgenes enhance seed survival also in the in the DS10:A9#6-7 genetic background. Click here for file

Additional file 3 **1D-electrophoresis analyses of the accumulation of HSPs and dehydrins in seeds of the DS10:A9/DR2 lines**. 1D-Western blot analyses of HSP and dehydrin accumulation showing only very subtle protein accumulation changes in seeds of the DS10:A9/DR2 lines. Click here for file

Additional file 4 **Unaltered, soluble, carbohydrate content in seeds of the DS10:A9/DR2 lines**. Total soluble carbohydrate is the same for DS10:A9 and DS10:A9/DR2 seeds. Click here for file

Additional file 5 **The *35S:DR2 *transgene when combined with *35S:A9 *does not further enhance tolerance to severe dehydration (analyzed by leaf survival) or accumulation of sHSPs**. Survival of green organs after severe dehydration in DT2 assays and the accumulation of sHSPs are not enhanced in the 35S:A9/DR2 plants. Click here for file

Additional file 6 **The predicted amino acid sequence of HaDREB2 shows unique features that are conserved in some DREB2 factors, but not in AtDREB2A**. Some sequence features of HaDREB2 are conserved only in some DREB2 factors from different plants not including Arabidopsis. Click here for file
