# Supplementary material for: The HaDREB2 transcription factor enhances basal thermotolerance and longevity of seeds through functional interaction with HaHSFA9
Source: BMC Plant Biol. 2009 Jun 19;9:75. doi: 10.1186/1471-2229-9-75 (PMC2706249; doi:10.1186/1471-2229-9-75)
Supplement: Additional file 1 — Accumulation of HSPs in seeds from DS10:DR2 lines to similar levels as in non-transgenic seeds. Western blot analyses of HSPs in seeds from DS10:DR2 lines showing that their accumulation levels are indistinguishable from negative controls. [file 1471-2229-9-75-S1.pdf]

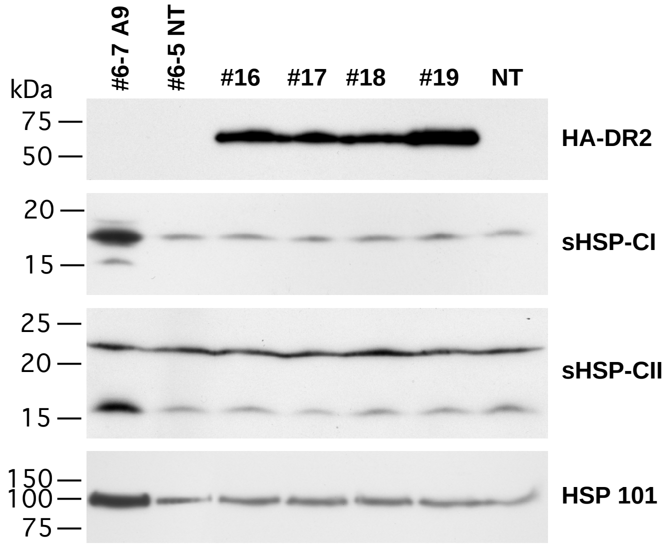

Additional file 1 *Accumulation of HSPs in seeds from DS10:DR2 lines to similar levels as in non-transgenic seeds.*

Western blot showing accumulation of HA-tagged HaDREB2 (HA-DR2) in the DS10:DR2 lines. Detection with antibodies against the HA tag. The accumulation of different HSPs (sHSP-CI, sHSP-CII and HSP101) was analyzed in protein samples of mature seeds from the analyzed lines. Protein accumulation levels were indistinguishable from negative controls: NT, non-transgenic tobacco, and #6-5 NT, a non-transgenic sibling line that segregated from line DS10:A9#6-7 (#6-7 A9). As previously reported (see reference 3 in the manuscript), line #6-7 A9 provided a positive control for enhanced accumulation of seed HSPs. The HSP-specific antibodies used for immunodetection are indicated on the right. Molecular mass markers (in kDa) are indicated on the left.
