# Supplementary material for: The HaDREB2 transcription factor enhances basal thermotolerance and longevity of seeds through functional interaction with HaHSFA9
Source: BMC Plant Biol. 2009 Jun 19;9:75. doi: 10.1186/1471-2229-9-75 (PMC2706249; doi:10.1186/1471-2229-9-75)
Supplement: Additional file 2 — Combined DS10:DR2 and DS10:A9 transgenes in homozygosis enhance seed survival after BTA: results in the DS10:A9#6-7 genetic background. BTA assays showing that combined DS10:DR2 and DS10:A9 transgenes enhance seed survival also in the in the DS10:A9#6-7 genetic background. [file 1471-2229-9-75-S2.pdf]

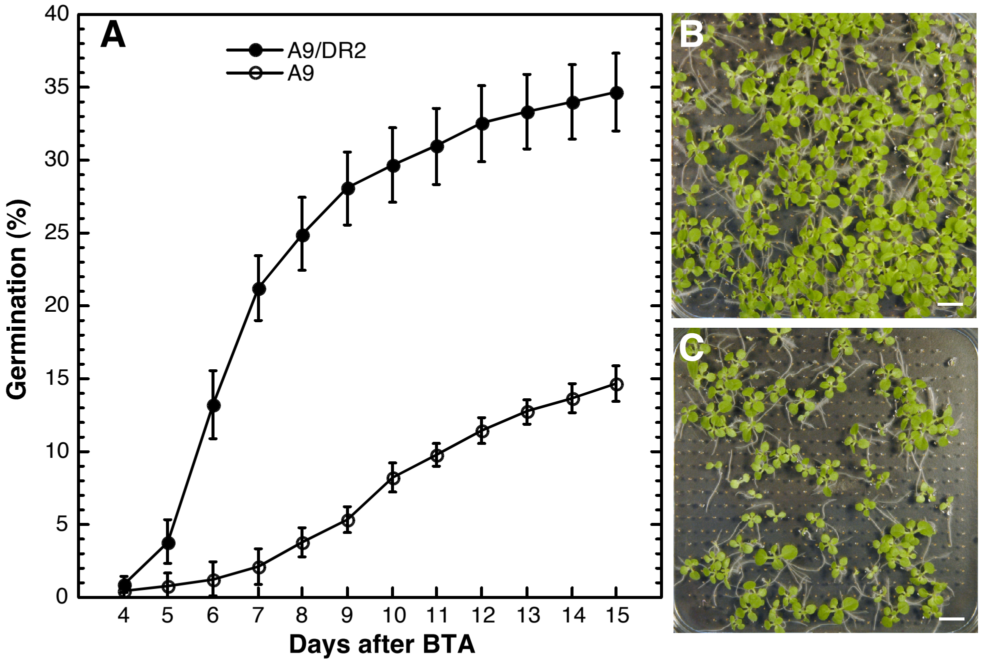

Additional file 2 *Combined DS10:DR2 and DS10:A9 transgenes in homozygosis enhance seed survival after BTA: results in the DS10:A9#6-7 genetic background.*

Germination after BTA of double-homozygous seeds (A9/DR2) compared with that of seeds from a sibling line without DR2 (A9), all in the DS10:A9#6-7 parental genetic background. A. - We show average germination, at different time after BTA, from 5 or 4 independent experiments respectively performed with line A9#6-7/DR2#25-2 and a sibling line without DR2: A9#6-7/#25-1. Statistical analyses confirmed highly significant differences between the germination percentages of single and double-homozygous seeds over the duration of the experiment (4-15 d after BTA [$F= 50.31$, $P= 0.0002$, 1 and 77 df; repeated-measures ANOVA]). We also show pictures of representative results of individual experimental samples, and showing germination 15 days after BTA: B. - A9#6-7/DR2#25-2 (A9/DR2). C. - A9#6-7/#25-1 (A9). Scale bars, 10 mm.
