# Supplementary material for: The HaDREB2 transcription factor enhances basal thermotolerance and longevity of seeds through functional interaction with HaHSFA9
Source: BMC Plant Biol. 2009 Jun 19;9:75. doi: 10.1186/1471-2229-9-75 (PMC2706249; doi:10.1186/1471-2229-9-75)
Supplement: Additional file 3 — 1D-electrophoresis analyses of the accumulation of HSPs and dehydrins in seeds of the DS10:A9/DR2 lines. 1D-Western blot analyses of HSP and dehydrin accumulation showing only very subtle protein accumulation changes in seeds of the DS10:A9/DR2 lines. [file 1471-2229-9-75-S3.pdf]

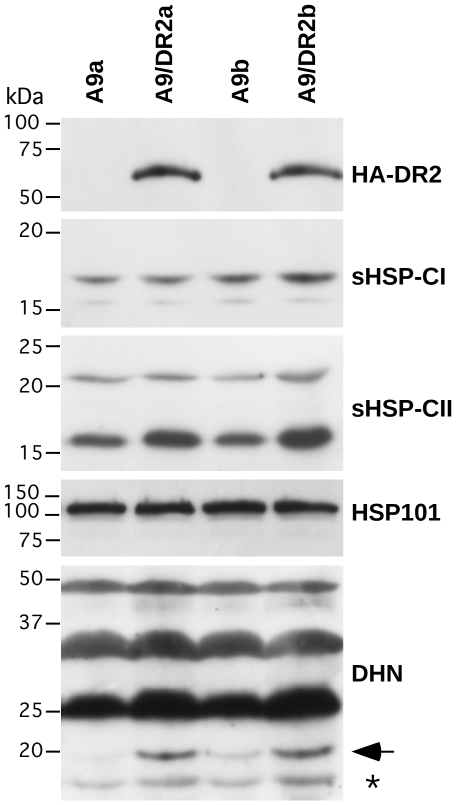

Additional file 3 *1D-electrophoresis analyses of the accumulation of HSPs and dehydrins in seeds of the DS10:A9/DR2 lines.*

1D Western analysis of seed protein samples from two, representative, double-homozygous DS10:A9/DR2 lines and from their sibling DS10:A9 lines, which respectively are: A9/DR2a (A9#14-5/DR2#23-5), A9/DR2b (A9#14-5/DR2#5-7), A9a (A9#14-5/#23-6), and A9b (A9#14-5/#5-4). The accumulation of different HSPs (sHSP-CI, sHSP-CII and HSP101), dehydrins (DHN), or HA-tagged HaDREB2 (HA-DR2) was detected using the specific antibodies indicated to the right. The arrow marks minor protein species that reacted with antibodies against plant dehydrins and that showed increased accumulation in the DS10:A9/DR2 lines. The asterisk marks a faint non-specific band detected with the same antibodies. Molecular mass markers (in kDa) are indicated on the left.
