# Supplementary material for: The HaDREB2 transcription factor enhances basal thermotolerance and longevity of seeds through functional interaction with HaHSFA9
Source: BMC Plant Biol. 2009 Jun 19;9:75. doi: 10.1186/1471-2229-9-75 (PMC2706249; doi:10.1186/1471-2229-9-75)
Supplement: Additional file 4 — Unaltered, soluble, carbohydrate content in seeds of the DS10:A9/DR2 lines. Total soluble carbohydrate is the same for DS10:A9 and DS10:A9/DR2 seeds. [file 1471-2229-9-75-S4.pdf]

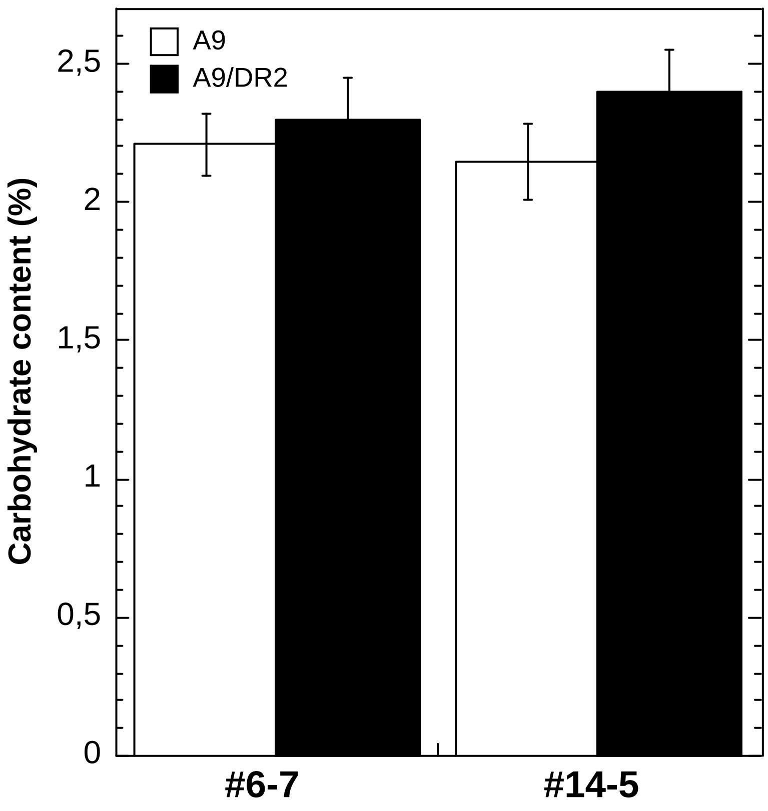

Additional file 4 *Unaltered, soluble, carbohydrate content in seeds of the DS10:A9/DR2 lines.*

Determination of, soluble, carbohydrate content in double-homozygous seeds (A9/DR2) compared with that of sibling lines without DR2 (A9). Comparisons were performed in the two parental genetic backgrounds: DS10:A9#6-7 (#6-7) and DS10:A9#14-5 (#14-5). We show average results from five independent experimental determinations. Each sample was measured 2 to 3 times. No significant difference, in carbohydrate content, was observed in either genetic background (for #6-7, F= 0.287 and P= 0.597; for #14-5, F= 2.133 and P= 0.158).
