# Supplementary material for: The HaDREB2 transcription factor enhances basal thermotolerance and longevity of seeds through functional interaction with HaHSFA9
Source: BMC Plant Biol. 2009 Jun 19;9:75. doi: 10.1186/1471-2229-9-75 (PMC2706249; doi:10.1186/1471-2229-9-75)
Supplement: Additional file 5 — The 35S:DR2 transgene when combined with 35S:A9 does not further enhance tolerance to severe dehydration (analyzed by leaf survival) or accumulation of sHSPs. Survival of green organs after severe dehydration in DT2 assays and the accumulation of sHSPs are not enhanced in the 35S:A9/DR2 plants. [file 1471-2229-9-75-S5.pdf]

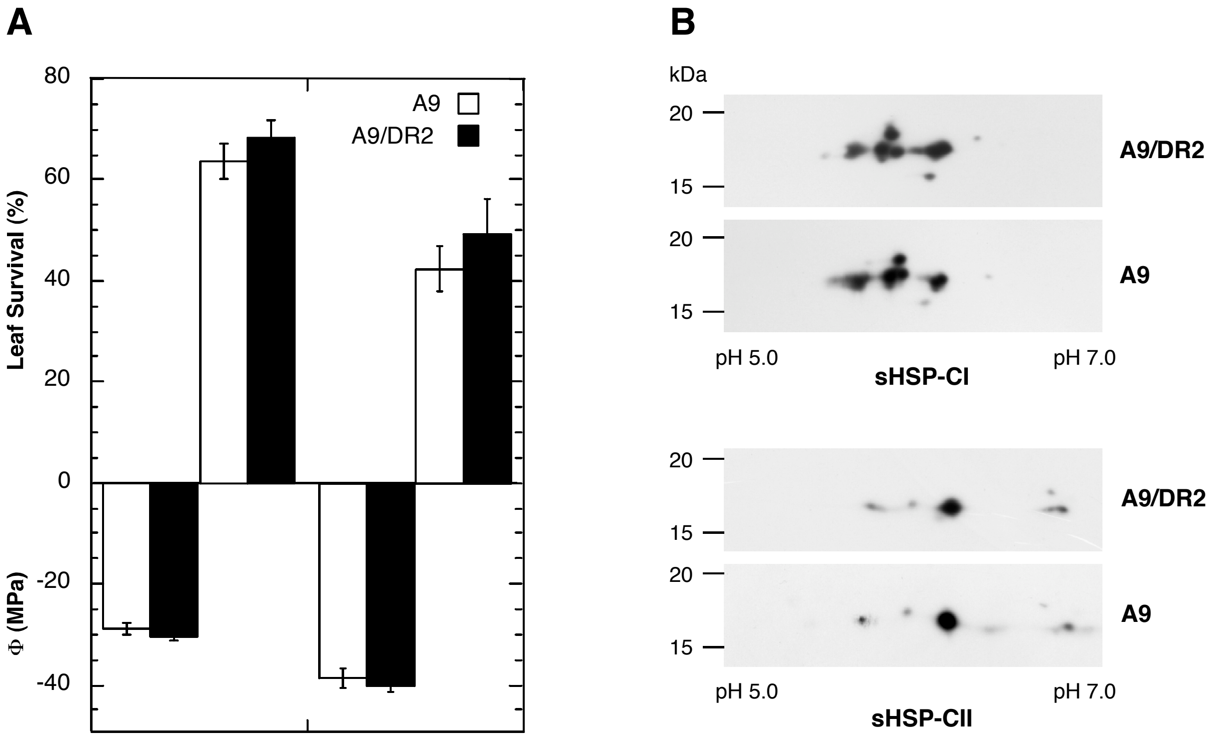

Additional file 5 *The 35S:DR2 transgene when combined with 35S:A9 does not further enhance tolerance to severe dehydration (analyzed by leaf survival) or accumulation of sHSPs.*

A.- Leaf survival was scored as the percentage of seedlings with green leave(s) that resisted DT2, even if the whole seedling did not resist (top). Average water potential (Φ) reached in the different DT2 assays (bottom). We used the same 35S:A9/DR2 lines (A9/DR2), and the corresponding, sibling, 35S:A9 lines (A9) employed for the experiments in Figure 5. Data were obtained from 3 to 14 experimental repeats per line and average water potential condition. B. - Representative sHSP-CI (top) and sHSP-CII (bottom) accumulation patterns determined by 2D-western analysis. We could not observe reproducible differences between A9/DR2 and A9 lines.
