# Supplementary material for: The HaDREB2 transcription factor enhances basal thermotolerance and longevity of seeds through functional interaction with HaHSFA9
Source: BMC Plant Biol. 2009 Jun 19;9:75. doi: 10.1186/1471-2229-9-75 (PMC2706249; doi:10.1186/1471-2229-9-75)
Supplement: Additional file 6 — The predicted amino acid sequence of HaDREB2 shows unique features that are conserved in some DREB2 factors, but not in AtDREB2A. Some sequence features of HaDREB2 are conserved only in some DREB2 factors from different plants not including Arabidopsis. [file 1471-2229-9-75-S6.pdf]

```
Ha  MALGFSDRNTEMSPYSQQSNTSAVPMDSSKKRKTRSRKEAPEKVAATLAKWIEYNKANE-  59
Dv  -----------MTNSDQPSTTPSSSMDSPRKRKSRSRRDGPKGVAETLAKWKEYNNKIDS  49
Le  -----------MAIMDEAANMVCVPLDYSRKRKSRSRRDRTKNVEETLAKWKEYNEKLDN  49
Cr  -----------MALLDQASNLSPMPFDFTRKRKSR-RRDGTKNVAETLAKWKEYNEKLDA  48
At  -----------MAVYDQSGDRNRTQIDTSRKRKSRSRGDGTT-VAERLKRWKEYNETVEE  48

Ha  -DGKAK-TRKAPAKGSKKGCMKGKGGPENSRTNFRGVRQRTWGKWVAEIREPNRGRRLWL  117
Dv  LDEKAKPARKVPAKGSKKGCMKGKGGPENSRCKFRGVRQRTWGKWVAEIREPNRGSRLWL  109
Le  -EGKGKPVRKVPAKGSKKGCMRGKGGPENWRCKYRGVRQRIWGKWVAEIREPKRGSRLWL  108
Cr  -LDGGKPARKVPAKGSKKGCMKGKGGPENSHCKYRGVRQRTWGKWVAEIREPNRGSRLWL  107
At  ---VSTKKRKVPAKGSKKGCMKGKGGPENSRCSFRGVRQRIWGKWVAEIREPNRGSRLWL  105

Ha  GTFGSAVEAALAYDEAARAMYGTSARLNLPNCRSKNDILPLLVTN-PASSCDSTTTCSYS  176
Dv  GTFGSAVEAALAYDEAARVMYGSCARLNLPNCRSMNEYSQMVVPNGSASSCDSTTTCSHS  169
Le  GTFGTAIEAALAYDDAARAMYGPCARLNLPNYACDSVSWATTSAS--ASASDCTVASGFG  166
Cr  GTFRNAIEAALAYDEAARAMYGPCARLNLPNYRASEE--SSSLPT--TSGSDTTTASGIS  163
At  GTFPTAQEAASAYDEAAKAMYGPLARLNFPRSDASEVTSTSSQSE--VCTVETPGCVHVK  163

Ha  EVDATHDS---------------------------KPGPAVFPSVKHEESVQVKHEP---  206
Dv  LEDSKASSS--------------------------RQMIKQDEGDSKREDTIVKHEVKEE  203
Le  EVCPVDG----------------------------------ALHEADTPLSSVKDEGTAM  192
Cr  EVSVYEDKKFTP-------VVSGLKQDDKGESLESADSKPQLLVDAGTPMSAVKEEPKEY  216
At  TEDPDCESKPFSGGVEPMYCLENGAEEMKRGVKADKHWLSEFEHNYWSDILKEKEKQKEQ  223

Ha  ------EIVAKEEHCVDN-------------------------------NDLGFDIGD--  227
Dv  PVEEFKSIRIKDEPVETN-------------------------------EDISLPHVN--  230
Le  DIVEPTS---IDEDTLKS-----------------------------GWDCLDKLNMD--  218
Cr  QVMDSQSEGQFGDEEPPSKLVCKEVDFGQDQAVVPAVKNAEEMGGEMGGDILKGCSLS--  274
At  GIVETCQQQQQDSLSVADYG---------------------------WPNDVDQSHLDSS  256

Ha  EMFDLEELLGAVEDSNPE------------------------------------AGSGDG  251
Dv  EMFDMEELLEMMGQRKSQNTGNRMGQNGFDEPSGVSQPWIKIEPQSEYQQDTYGTGGGFG  290
Le  EMFDVDELLAMLDSTPVFTK----------------------------------DYNSDG  244
Cr  EMFDVDELLSVLDSTPLHAS----------------------------------DFQHGM  300
At  DMFDVDELLRDLNGDDVFAG-----------------------------------LNQDR  281

Ha  YDGRFVN-----------GADANENMQMYMQP---DPCVSG--QDYSFDFLQPGRPEDCS  295
Dv  FDFSMPKQPAMDQKLGDMGWFDQEPMNPYTRPKWEDKCDVGAVADYGFDFLMPGRPEDGN  350
Le  KHNNMVSDS--------------QCQEP-------NAVVDPMTVDYGFDFLKPGRQEDLN  283
Cr  GNGNVKAEAAYNYAPSWDSAFQLQNQDPKLGSQ-QHMAQTPPEINSGIDFLQPGRQEDSY  359
At  YPGNSVAN----------GSYRPESQQS--------GFDPLQSLNYGIPPFQLEGKDGNG  323

Ha  FTLEELG-LGLDAELDLYSN  314
Dv  FTLQELG-FDLGADFGL---  366
Le  FSSDDLAFIDLDSELVV---  300
Cr  FTLGDLDFLDLGAELGL---  376
At  F-FDDLSYLDLEN-------  335
```

Additional File 6 The predicted amino acid sequence of HaDREB2 shows unique features that are conserved in some DREB2 factors, but not in AtDREB2A.

Alignment, using the Clustal X program [Larkin et al., Bioinformatics 23(21): 2947-2948 (2007)], of the predicted HaDREB2 protein (Ha) with other subgroup A-2 DREB proteins [9]: DvDREB2A (ABR23508, Dv), LeDREB1 (AAN77051, Le), CrORCA1 (CAB93939, Cr), and AtDREB2A (At). The highly conserved AP2 domain is underlined. Within the AP2 domain, the Valine-14 and Aspartic acid-19 residues characteristic of DREB proteins [9] are indicated by dots. Some AP2 domain residues conserved in the HaDREB2, DvDREB2A, LeDREB1, and CrORCA1 proteins, but not in AtDREB2A, are indicated by filled triangles. On the AtDREB2A protein, and next to AP2 domain, the sequences (amino acids 136 to 165 [18]) involved in its instability are boxed. These sequences are not present in HaDREB2, DvDREB2A, LeDREB1, or CrORCA1 proteins. The carboxyl-

terminal region is less conserved and enriched in acidic residues that could participate in transcriptional activation [10, 18]. On the alignment we indicate a carboxyl-terminal motif that is conserved in all five proteins (box with thin line). This motif is included in the sequences required for transcriptional activation by AtDREB2A in protoplast assays; the 81-amino acid, terminal, fragment between positions 254 and 335 in AtDREB2A [18]. Similarly, transcription activation by ZmDREB2A required the carboxyl-terminal 83 amino acids. In this case the necessary fragment included amino acid motifs specific for mono-cot DREB2 proteins [14]. We also indicate a second motif conserved in the HaDREB2, DvDREB2A, LeDREB1, and CrORCA1 proteins, but not in AtDREB2A (box with thick line). Therefore, DREB factors from different plant groups could use dissimilar motifs for transcriptional activation; these motifs would be located in the carboxyl-terminal 80-90 amino acids. The conserved amino acid residues are highlighted with black (when identical) or gray (when similar) background. Dashes show gaps in the amino acid sequences introduced to optimize alignment.
